# Scrub Typhus Meningoencephalitis: Review of Literature and Unique Diagnostic & Management Challenges in Resource-Limited Settings

**DOI:** 10.7759/cureus.26369

**Published:** 2022-06-27

**Authors:** Susmita Unni, SK Chellapandian Eswaradass, Hari Krishnan Nair, Swapna Anandan, Iswariya Mani, Prasanna Venkatesan Eswaradass

**Affiliations:** 1 Medicine, St. George's University, St. George's, GRD; 2 Neurology, Appusami Hospital, Salem, IND; 3 Medicine, Saint Louis University School of Medicine, St. Louis, USA; 4 Medicine, Yale New Haven Hospital, New Haven, USA; 5 Endocrinology, UPMC Children's Hospital, Pittsburgh, USA; 6 Neurology, University of Tennessee Medical Center, Memphis, USA

**Keywords:** chigger, zoonotic disease, public health, india, leptotrombidium, trombiculid, orientia tsutsugamushi, doxycycline, meningoencephalitis, scrub typhus

## Abstract

Scrub typhus is a zoonotic febrile illness caused by* Orientia tsutsugamushi* and transmitted by *Leptotrombidium* larvae. Scrub typhus often presents with nonspecific clinical features, and ranges in severity from mild illness to multiorgan failure and fatality. The disease is primarily found in the Asia-Pacific rim, including India, Pakistan, Thailand, Malaysia, Korea, and China. Due to frequent limitations in healthcare resources in many of these countries, the diagnosis and management of scrub typhus meningoencephalitis pose unique challenges. This review focuses on the epidemiology, clinical features, diagnostic testing, and management modalities in such resource-limited settings. Exercising a high index of clinical suspicion and timely diagnostic tests and management strategies are vital to prevent life-threatening complications of this treatable illness.

## Introduction and background

Introduction

Scrub typhus is a zoonotic infectious disease caused by the Gram-negative coccobacillus *Orientia tsutsugamushi*. The genus *Orienta* is classified under the order Rickettsiales within the family Rickettsiaceae. It is transmitted to human beings by the bite of vectors such as hard or soft ticks, fleas, mosquitoes, mites, and lice. Scrub typhus is specifically transmitted by the larvae of trombiculid mites of the genus *Leptotrombidium* [1]. The larval trombiculid mites, also called chiggers, are considered both a vector for scrub typhus and a reservoir for *O. tsutsugamushi*. Scrub typhus often presents insidiously with non-specific clinical features and the severity of infection can range from mild signs and symptoms to multiorgan failure and death [2]. Meningitis and encephalitis syndromes are known complications of the disease and are reported in the literature with varying frequencies [3].

Epidemiology

Approximately one billion people are at risk for scrub typhus and about one million new cases are identified annually. The primary distribution of the disease is the Asia-Pacific rim, with the organism being endemic in countries such as India, Pakistan, Thailand, Malaysia, Korea, and China [4]. Scrub typhus has been reported as early as the 1930s in India, with outbreaks occurring among troops during World War II [5]. Being a disease predominantly distributed in countries that are traditionally classified as resource-limited, the diagnosis and management of scrub typhus meningoencephalitis pose unique challenges and necessitate the adoption of novel strategies.

## Review

Periodic outbreaks of this zoonotic illness have occurred across India. One community-based study showed that scrub typhus and other rickettsial diseases were widely distributed in the southern Indian state of Tamil Nadu [6]. However, the non-specific clinical features, lack of specific diagnostic tools, and low index of suspicion by physicians have resulted in its underreporting and undertreatment [7,8].

Clinical features and complications

Scrub typhus presents clinically as a nonspecific febrile illness accompanied by headache, anorexia and malaise, nausea, vomiting, diarrhea, cough, or breathlessness. This may progress to chills and fever by the third or fourth day of the bite, and a non-pruritic maculopapular rash and lymphadenopathy may appear by the end of the first week. In addition, some patients develop an eschar at the site of the chigger bite, which is a pathognomonic clinical sign. The prevalence of eschars varies from 20% to 86% as reported in various studies [2]. The identification of eschar is quite difficult in the Indian population due to darker skin tone. It is interesting to note that a study by Lee et al. showed that the absence of eschar was associated with increased mortality [3].

The organism also induces vasculitis leading to symptoms of systemic organ invasion during the second week of illness, including pneumonitis, pleural effusion, myocarditis, acute kidney injury, acute respiratory distress syndrome, meningitis, and encephalitis [2,9-14].

Central nervous system (CNS) involvement in scrub typhus manifests in myriad clinical patterns, including meningoencephalitis, cerebral infarction, cerebellitis, cranial nerve palsies, plexopathy, transverse myelitis, neuroleptic malignant syndrome, and Guillain-Barré syndrome, and these patients may present with seizures, neck stiffness, altered level of consciousness, delirium, and coma [15,16]. There have also been reports of scrub typhus patients presenting clinically with opsoclonus-myoclonus, transient parkinsonism, and pain similar to trigeminal neuralgia [17-19].

Meningoencephalitis may be seen in two-thirds of patients with scrub typhus, and a high suspicion should be maintained in patients from endemic regions presenting with febrile encephalopathy. Meningeal involvement in scrub typhus can range from aseptic meningitis to frank meningoencephalitis [20,21]. Although the meningoencephalitis associated with scrub typhus usually presents without any focal neurological signs, bilateral sixth and seventh cranial nerve palsies have been reported [22].

The CNS manifestations of scrub typhus are due to small-vessel vasculitis, causing a secondary breakdown in the blood-brain barrier and leading to cerebral microinfarctions and edema [23]. The organism directly invades the cerebrospinal fluid and has been seen to grow in the cerebrospinal fluid previously [24]. Using nested polymerase chain reaction (PCR), rickettsial DNA has been isolated in cerebrospinal fluid (CSF) [25]. A prospective study conducted on a group of 30 children in Thailand revealed scrub typhus to be the second-most common cause of aseptic meningitis, next to Japanese encephalitis [26].

Diagnosis

The Weil-Felix test is a serological test based upon the cross-reaction between anti-rickettsial antibodies and Proteus antigens (OX2 and OX19). In a study conducted by Eswaradass et al., the epidemiologic, clinical, and laboratory profiles of 15 patients admitted to a tertiary care hospital in south India, were identified. A titer of >1:80 in the Weil-Felix test was defined as positive after ruling out malaria, dengue, leptospirosis, and bacterial, viral, and tuberculous meningitis, which are other common diseases prevalent in the region. All patients had the triad of fever, altered sensorium, and meningeal signs. Headache, myalgia, and seizures were seen in 86%, 93%, and 66.6%, respectively. Two patients had hemiparesis, and eschar was seen in three patients. The most common lab abnormalities were elevated liver enzymes (46%), renal failure (26%), and thrombocytopenia (13%). Cerebrospinal fluid was abnormal in all patients, with protein elevation (66.6%), lymphocytic pleocytosis (100%), and hypoglycorrhachia (40%). Weil-Felix test was strongly positive with a titer of > 1:160 in 86% of patients. Four patients developed acute respiratory distress syndrome (ARDS). There was an excellent response to doxycycline [27].

In a study conducted in south India, the sensitivity for OX-K was 30% at a titer breakpoint of 1:80, but the specificity and positive predictive value were 100% [7]. Weil-Felix test results may be negative during the early stages of the disease because agglutinating antibodies are detectable only during the second week of the illness. Moreover, early treatment of the disease can blunt the serological response. Despite its drawbacks, the Weil-Felix test still serves as a useful and the most cost-effective tool available for the laboratory diagnosis of rickettsial diseases. Hence it is still acceptable in a developing country like India where the disease remains largely under-recognized and where availability and the cost of other investigative modalities are major issues, and it is suggested that the diagnosis of scrub typhus should be largely based on a high index of suspicion and careful clinical, laboratory, and epidemiological evaluation [28].

In a study conducted by Oberoi et al. in a tertiary care hospital in India, out of 98 patients who tested positive for antibodies against *O. tsutsugamushi*, all had a persistent high-grade fever, and eschar was seen in 10.2% of the patients [29]. In the previously-mentioned study conducted by Eswaradas et al., all patients had the triad of fever, altered level of consciousness, and meningeal signs [27]. Based on several previous clinical studies, routine laboratory investigations in patients with scrub typhus have revealed early lymphopenia with late lymphocytosis, a decrease in the ratio of the cluster of differentiation 4 (CD4):CD8 lymphocytes, and thrombocytopenia [30]. Transaminases are elevated in most patients, and about half of the patients demonstrate hypoalbuminemia, which is thought to be from perivasculitis and endothelial damage leading to capillary leak [31,32].

Silpapojakul et al., in a case series of nine patients who presented with scrub typhus and three patients who presented with murine typhus meningitis and/or meningoencephalitis, reported a predominantly mononuclear CSF pleocytosis with lower total WBC counts (<150 cells/cu mm) [23]. Pai et al. conducted a study on 25 patients with scrub typhus having CNS involvement, and half of them had CSF lymphocytosis, and one-third had elevated protein levels [25]. Similar results were reported in another retrospective study conducted by Viswanathan et al. [33]. In contrast, in the previously-mentioned study by Eswaradass et al., CSF was abnormal in all patients with elevated protein levels (66.6%), lymphocytic pleocytosis (100%), and hypoglycorrhachia (40%) [27]. These findings can be confused with tuberculous meningitis which is very common in developing countries such as India. However, initiating empiric anti-tuberculosis therapy (ATT) in these patients may worsen the liver function. In the same study by Eswaradas et al., 46% of patients had abnormally elevated liver enzymes which could potentially worsen with the initiation of empiric ATT, and hence a strong clinical suspicion for scrub typhus must be present for the initiation of scrub typhus-directed antimicrobial therapy [27].

Treatment

Doxycycline (100 mg orally or intravenously twice daily) is currently the drug of choice for this illness and has proven its efficacy through several clinical trials. However, instances have been reported in literature where progressive neurological damage has occurred despite treatment with doxycycline. The reasons for these are either drug resistance, difficulty in penetrating the blood-brain barrier, or immune-mediated injury. The use of antimicrobial therapy with tetracycline and chloramphenicol should be avoided in children and pregnant women because of the risk of adverse effects and teratogenicity, respectively. Macrolides, quinolones, and rifampin are also used in the treatment of scrub typhus [34].

## Conclusions

Scrub typhus is a disease that is frequently underdiagnosed in resource-limited countries including the Indian subcontinent due to the nonspecific clinical features that it presents with and the limited availability of appropriate diagnostic facilities. Physicians must be wary of its possible development by being vigilant about its symptoms and laboratory findings. Patients who present with meningoencephalitis and altered liver function tests should raise a high index of suspicion for scrub typhus. Eschar may give a clue to diagnosis but is of limited value as it is present only in 20% to 30% of the patients, and frequently goes unnoticed in people with darker skin. Scrub typhus meningoencephalitis can mimic tuberculous meningitis and starting antitubercular treatment can worsen liver function. Exercising a high degree of clinical suspicion and initiating timely treatment with doxycycline is necessary to prevent life-threatening complications.
